# Cholera in Sub-Saharan Africa: Unveiling neglected drivers and pathways to elimination

**DOI:** 10.1371/journal.pntd.0013029

**Published:** 2025-04-23

**Authors:** Beenzu Siamalube, Emmanuel Ehinmitan, Steven Runo, Maina Ngotho, Justus Onguso

**Affiliations:** 1 Department of Molecular Biology and Biotechnology, Pan African University Institute for Basic Sciences, Technology and Innovation, Nairobi, Kenya; 2 Department of Biochemistry, Microbiology and Biotechnology, Kenyatta University, Nairobi, Kenya; 3 Department of Animal Sciences, Jomo Kenyatta University of Agriculture and Technology, Nairobi, Kenya; 4 Institute for Biotechnology Research, Jomo Kenyatta University of Agriculture and Technology, Nairobi, Kenya; Universidad Nacional Autonoma de Mexico, MEXICO

## Abstract

Cholera is a virulent infectious disease caused by the Gram-negative, comma-shaped bacteria *Vibrio cholerae*, after ingesting contaminated food and/or water. If left untreated, it can kill within 5 days. Since mid-2021 the world has recorded a notable increase in the seventh cholera pandemic, with high case fatality rate especially in Sub-Saharan Africa. Oral cholera vaccines are established but not readily available on the market, or if they are, they are not pocket friendly for low-resource-income countries. Hence, with the advent of green factory biotechnology, plant-derived edible vaccines are such a promising approach to supplement conventional vaccine methods. Human travellers are often the major transmitters as they move from region to region. Poor sanitation and inadequate clean water supply are services not readily available in most Sub-Saharan African countries, coupled with insufficient surveillance services, lack of early detection facilities, and the public not having ample awareness concerning sanitation and hygiene. This article highlights the epidemiology of cholera in Africa and expounds on what drives the outbreaks of cholera in this region. The discussion provides an in-depth analysis of the factors leading to the forsaken cholera drivers, emphasizing economic factors, culture, and environmental influences, particularly within the Sub-Saharan African communities. It presents a strategic blueprint approach that includes public health awareness, community participation, government involvement, and exploring emerging research tools. By merging these proposals into a unified context, a collective and practical methodology would be established to tackle the impact of cholera epidemiology that has been sidelined in Sub-Saharan Africa.

## 1. Introduction

Cholera remains a significant public health challenge in Sub-Saharan Africa (SSA), disproportionately affecting vulnerable populations with limited access to clean water, sanitation, and healthcare. Despite decades of research and intervention efforts, outbreaks persist, exacerbated by environmental, epidemiological, and socio-political factors [1]. This article aims to critically examine the drivers of cholera in SSA and propose a comprehensive roadmap toward its elimination. By addressing gaps in the existing literature and integrating recent research findings, this viewpoint contributes to the discourse on effective cholera prevention and control [2].

### 1.1. Geographical epidemiology of cholera

Cholera is endemic in many parts of SSA, with recurrent outbreaks fuelled by climate change, population displacement, and inadequate public health infrastructure. According to recent studies [2], the region has witnessed fluctuating case numbers over the past two decades, with notable peaks linked to extreme weather events and socio-political instability. From 2010 to 2019, the World Health Organization (WHO) recorded over 1,080,778 cholera cases in SSA, out of the cumulative 4,426,844 cases. The region accounted for 24% of the global cases during the study period, contributing significantly to the global burden of the disease [3].

**Fig 1:**
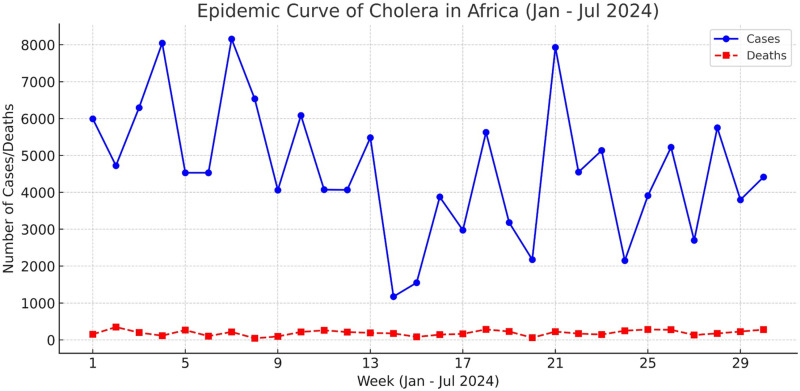
Weekly epidemic curve of cholera in Africa (January–July 2024). The figure illustrates the weekly number of reported cholera cases and deaths in Africa from January to July 2024. The solid blue line represents the number of cholera cases, while the dashed red line represents the number of deaths. The trend highlights fluctuations in disease incidence over time, providing insights into the progression of the outbreak [6].

The seventh cholera pandemic, ongoing since 1961, has persisted in SSA due to inadequate sanitation, contaminated water sources, and insufficient public health interventions. Cholera outbreaks in SSA follow seasonal patterns, with peaks during rainy seasons due to increased water contamination [4]. From January to July of 2024, fourteen (14) SSA countries reported cholera outbreaks, with 112,301 cases and 1,900 deaths. Zimbabwe, Ethiopia, and the Democratic Republic of Congo recorded over 20,000 cholera cases each, whereas South Africa, Cameroon, and Uganda each reported cases below 100. Weeks 4, 7, and 21 recorded the highest number of cholera cases (~8,000), while week 14 had the least with up to 1,000 cases recorded (Fig 1). The total case fatality rate was 1.7%, notably above the WHO-recommended 1% threshold, indicating the ineffectiveness of current prevention strategies [5].

### 1.2. Clinical epidemiology of cholera

*Vibrio cholerae*, the causative agent of cholera, is a Gram-negative, facultative anaerobic bacterium found in aquatic environments. It exists in multiple serogroups, with O1 and O139 being the most significant in cholera outbreaks [7]. It thrives in brackish water and can acquire virulence traits under environmental stress. The cholera toxin (CT) is a major virulence factor of *V. cholerae* and is encoded by the ctxAB operon, which is carried by the CTXϕ bacteriophage [8]. CTXϕ plays a crucial role in CT production by transferring and regulating ctxAB gene expression. Environmental factors, such as pH, temperature, and quorum sensing signals dynamically regulate toxin expression, ensuring *V. cholerae* adapts efficiently to both aquatic reservoirs and human hosts [9].

Cholera transmission occurs primarily through the ingestion of contaminated water and food, with risk factors including poor sanitation, lack of clean drinking water, and overcrowded living conditions [5]. Clinical manifestations range from mild diarrhoea to severe dehydration, which, if left untreated, can result in death within hours [10]. Management strategies include oral rehydration therapy, intravenous fluids, and antibiotic treatment in severe cases. Prevention and control measures focus on improving water, sanitation, and hygiene (WASH) infrastructure, and vaccination campaigns. Likewise, community-based interventions, such as the provision of safe drinking water or sensitizing the people about treating drinking water by boiling or chlorination, could be 'implemented' [11]. Furthermore, advise the people to wash their fruits and vegetables before consumption, as well as clean their hands before handling food and after visiting the toilet [12].

### 1.3. Justification and aims of the study

Despite ongoing efforts to control cholera, SSA continues to experience recurrent outbreaks. Existing research primarily focuses on epidemiological and medical aspects, with limited attention to ecological and socio-political factors influencing transmission [13]. This study seeks to address these knowledge gaps by providing a comprehensive analysis of cholera drivers in SSA, emphasizing environmental, economic, and governance-related determinants. Additionally, it explores innovative approaches, such as plant-derived edible vaccines (ECVs), to supplement current prevention strategies. Furthermore, the study aims at evaluating current prevention and control measures, including vaccine accessibility and proposing a strategic roadmap for sustainable cholera elimination in SSA [14].

## 2. Drivers of cholera transmission in Africa

### 2.1. Climate and hydrological factors

SSA’s tropical climate influences cholera dynamics, with outbreaks commonly occurring during wet seasons [15]. However, exceptions exist, such as Mozambique’s 2018 dry-season outbreak, likely driven by water shortages [16]. Research highlights a correlation between long-term climate trends and cholera incidence, necessitating improved predictive modelling for outbreak preparedness. Climate variability, including rising temperatures, fluctuating rainfall patterns, and extreme weather events like cyclones, has been linked to increased cholera outbreaks [17]. Changes in water salinity and pH, coupled with inadequate drainage systems, create optimal conditions for *V. cholerae* proliferation and transmission.

### 2.2. WASH coverage in Africa

Lack of sustainable access to clean water and sanitation is one of the key drivers of cholera transmission in Africa [18]. Many SSA countries [19–21] struggle to provide adequate sewage treatment and waste management, leading to the contamination of drinking water sources [22,23]. The widespread lack of adequate sewer treatment facilities in numerous African countries presents a major obstacle to effective wastewater management [24]. For example, in Ethiopia’s capital, Addis Ababa, less than 3% of wastewater is processed at treatment facilities. The Kaliti treatment plant, originally built in 1982 to serve 50,000 people, was only accommodating 13,000 individuals nearly three decades later, primarily due to limited household connections to the municipal sewer system [21]. Similarly, in Kenya’s Kisumu district, pump station failures lead to sewage overflow at upstream access points, causing untreated sewage to flow directly into Lake Victoria [25].

In Bamako, Mali, the absence of a sewer network forces approximately 80% of residents to rely on on-site sanitation facilities, as securing land for treatment sites remains a challenge [23]. Likewise, Kigali, Rwanda, lacks both a centralized sewer system and a main sewage treatment plant [20]. Most sewage disposal in the city relies on septic tanks with soak-away pits and pit latrines, with only a handful of semi-centralized treatment plants operating in specific areas [26]. Surveillance studies including a geospatial analysis of the public health risk posed by cholera in Lusaka, Zambia, informed improved water and sanitation provision [27]. Furthermore, educating the community on matters of personal hygiene and emphasizing the need for sustainable infrastructure investments, especially in SSA [24], would heighten access to basic WASH facilities [28]. Consequently, adhering to these measures would contribute largely to the fight against cholera and indeed other waterborne diseases, globally [29].

### 2.3. Human mobility and socioeconomic conditions

Population displacement due to conflict, economic migration, and urbanization exacerbates cholera transmission. Displaced populations often lack access to essential healthcare services, increasing their vulnerability to infectious diseases. Additionally, poverty and limited healthcare infrastructure hinder timely outbreak response [30]. Thus, adaptative healthcare interventions in migration-prone areas and leveraging social protection programs could enhance outbreak response.

### 2.4. Vaccine accessibility and limitations

Oral cholera vaccines (OCVs) are key tools in cholera prevention, especially Dukoral, Shanchol, and Euvichol – which are composed of whole cell killed *V. cholerae* O1 and O139 without toxin B subunit, except for Dukoral which has recombinant cholera toxin B subunit [31]. Their deployment depends on the outbreak situation, endemic risk, and available supply from WHO stockpiles [32]. OCVs have proven effective in short-term outbreak containment but face challenges, such as high production costs, limited shelf life, and inadequate distribution, especially in hard-to-reach areas [33]. Plant-derived ECVs offer a promising approach, as they would provide a stable, near-user-site, and cost-effective solution. ECVs are plant candidates that are genetically engineered to deliver antigenic proteins (i.e., cholera toxin B subunit) to the human immune system when ingested and protect the body against the invasion of *V. cholerae* [34]. A rice-based ECV was developed by scientists at the University of Tokyo, Japan. It underwent a randomized clinical trial, where it was proven safe and efficient in phase I of human subjects [35]. However, this biotechnique still requires further research and more clinical trials before widespread implementation, particularly in low- and middle-income countries like those of SSA.

### 2.5. Gaps in surveillance and response systems

Many SSA countries lack robust disease surveillance systems, leading to delayed outbreak detection and inadequate response mechanisms. Strengthening these systems through real-time data collection and integration of modern technologies, such as geographic information systems (GIS), is essential in the quest to combat cholera [36].

## 3. Roadmap to cholera elimination in Africa

### 3.1. Strengthening surveillance and early detection

To achieve cholera elimination, SSA countries must enhance disease monitoring systems. Investments in rapid diagnostic tools and community-based reporting mechanisms are crucial for timely outbreak response. GIS mapping and machine learning models can aid in predicting high-risk areas [37]. Similarly, developing real-time surveillance systems, such as artificial intelligence-driven predictive modelling [38] to detect and respond to cholera outbreaks quickly [36]. And strengthening cross-border collaboration [39] for disease monitoring among migrant and refugee populations [40] would fast-track the roadmap to cholera elimination in Africa.

### 3.2. Policy and governance reforms

Government commitment is essential for cholera control. Policymakers should adopt evidence-based strategies, including improved water governance and public–private partnerships. Case studies from regions that have successfully reduced cholera incidences, such as Haiti [41], can inform best practices. Establishing mobile health clinics and deploying healthcare workers to displacement camps would ensure disease containment in informal settlements. Additionally, integrating cholera prevention, vaccination, and treatment services into refugee and migrant health programs would be an ideal preventive measure to explore [42]. Also, upgrading and expanding water and sanitation infrastructure in informal settlements and underserved urban areas [43]. Increasing funding for healthcare systems in resource-limited settings to improve outbreak preparedness and response capacity [44]. Alongside implementing livelihood programs to reduce poverty [45] and improve living conditions in high-risk areas, would be brilliant governance reforms towards cholera elimination in Africa [46]. Strengthening policies that promote equitable access to healthcare for migrants and displaced individuals would be a game changer in ensuring that cholera incidences are reduced in the region [47].

### 3.3. Community engagement and public health awareness

Educational campaigns on hygiene and safe water practices can significantly reduce cholera episodes. Community-led initiatives, such as household water treatment and sanitation improvements, play a crucial role in behaviour change [48]. Thus, they should be prioritized. Besides, engaging local leaders, stakeholders, and leveraging social media platforms can enhance outreach efforts and the effectiveness of these interventions [49]. Furthermore, promoting public health awareness to all people of the community, such as presenting the WASH strategy in native and sign languages to enable everyone to grasp the concept seamlessly, as well as in braille to carter for individuals with special needs [50].

### 3.4. Advancing vaccine research and deployment

The use of OCVs has proven effective in outbreak control and prevention [51]. While emerging plant-derived edible vaccines hold promise, their efficacy in SSA settings remains untested. The development of edible vaccines from African crop varieties could be accelerated to address current traditional vaccine limitations. Alternatively, SSA countries could collaborate with global health organizations to establish local vaccine production facilities, ensuring equitable access. Prioritizing the deployment of WHO-approved OCVs [52] while exploring innovative vaccine solutions is crucial. Prioritizing OCVs in high-risk areas, including refugee camps and densely populated urban slums, and implementing routine vaccination programs alongside emergency response efforts would ensure wide coverage of vaccine deployment [53].

### 3.5. Strengthening WASH infrastructure

Investments in clean water supply, improved sanitation, and hygiene promotion are fundamental to cholera prevention. Lessons from successful interventions in countries like Bangladesh [54] can inform strategies tailored to SSA’s unique challenges. Additionally, providing displaced communities with immediate access to clean water, adequate sanitation, and hygiene facilities through solar disinfection facilities, mobile treatment units, emergency water supply systems, and temporary latrines [55]. Coupled with distributing hygiene kits, including soap, water purification tablets, and oral rehydration solutions, to displaced populations could help control the spread of cholera [56].

## 4. Successful cholera control models and their applicability in SSA

### 4.1. Bangladesh’s comprehensive cholera control model

Bangladesh implemented key strategies to control cholera outbreaks [54]. These included large-scale vaccination efforts targeting high-risk populations [31], promotion of tubewell-based drinking water [57], latrine construction, and behavioural change campaigns [58], alongside real-time disease tracking with mobile technology and laboratory-based diagnostics [59].

SSA can equally adapt these approaches by conducting OCV campaigns in cholera-endemic areas, prioritizing high-risk populations, such as urban slum dwellers and displaced communities [60]. The region can also establish decentralized water purification systems and low-cost filtration technologies in rural and peri-urban areas. As well as strengthen cholera surveillance through mobile health (mHealth) platforms and community-based reporting networks [61].

### 4.2. Haiti’s integrated cholera response (post-2010 outbreak)

Haiti in the Region of the Americas initiated rapid deployment of treatment centres [62], clean water supply [63], and emergency vaccinations [64]. The Caribbean nation equally trained community health workers to spread awareness and identify cases early. Additionally, Haiti committed to long-term investments to improve the WASH infrastructure [65].

African countries facing cholera outbreaks, could draw some lessons from Haiti and develop rapid response teams trained to contain outbreaks in high-risk areas [41]. Establishing community health worker networks could improve early case detection and promote preventive measures [66]. And securing long-term government and donor commitments for standard WASH infrastructure projects that can withstand unforeseen circumstances like natural disasters [67].

### 4.3. Zambia’s multi-sectoral approach to cholera control (2017–2018 outbreak)

Zambia in southern Africa set up a government-led cholera task force that coordinated efforts between ministries, non-governmental organizations, and international partners [68]. Furthermore, the landlocked country deployed emergency vaccination campaigns to high-risk populations [52] and enforced strict hygiene regulations for food markets and street vendors [69].

The other countries in the region could also establish national cholera task forces with clear roles for health ministries, water authorities, and local governments [70]. As well as expand OCV stockpiles for rapid deployment during outbreaks [71] and implement strict hygiene monitoring and licensing systems for informal food and water vendors to prevent cholera outbreaks [72].

### 4.4. Yemen’s use of artificial intelligence (AI) in cholera surveillance

Yemen enacted the use of AI-driven models to identify high-risk zones before outbreaks occur [73] and data-driven predictions to deploy medical supplies and clean water interventions efficiently [74].

Though resources could be limited in most SSA countries [22], AI-based cholera risk mapping using local climate, mobility [75], and infrastructure data could be a great preventive measure to help curb cholera outbreaks in the region [76]. This can be achieved by partnering with tech companies and universities [77] to integrate predictive modelling into national cholera response plans [78]. And training local health authorities to interpret and act on predictive analytics for outbreak prevention [42].

These models illustrate that successful cholera control requires a multi-sectoral approach integrating vaccination, WASH improvements, surveillance, policy reforms, and community engagement. By adapting these evidence-based interventions, SSA can develop a more effective, sustainable roadmap to cholera elimination [79].

## 5. Conclusion

Cholera remains a persistent threat in SSA, driven by environmental, social, and economic factors. While global elimination efforts are underway, significant gaps in surveillance, water access, and vaccine availability hinder progress. Addressing these challenges requires a multifaceted approach that includes improved disease monitoring, policy reforms, community engagement, and innovative vaccine strategies. To eradicate cholera, SSA must prioritize sustainable interventions, leveraging both traditional public health measures and emerging technologies. Collaborative efforts between governments, researchers, and international agencies are essential in making cholera a disease of the past in the region.
